# Investigations of Mechanisms Leading to Capacity Differences in Li/Na/K‐Ion Batteries with Conversion‐Type Transition‐Metal Sulfides Anodes

**DOI:** 10.1002/advs.202410653

**Published:** 2024-11-04

**Authors:** Kunxiong Zheng, Yongbiao Mu, Meisheng Han, Jie Liu, Zhiyu Zou, Hengyuan Hu, Youqi Chu, Fenghua Yu, Wenjia Li, Lei Wei, Lin Zeng, Tianshou Zhao

**Affiliations:** ^1^ Shenzhen Key Laboratory of Advanced Energy Storage Department of Mechanical and Energy Engineering Southern University of Science and Technology Shenzhen 518055 China; ^2^ SUSTech Energy Institute for Carbon Neutrality Southern University of Science and Technology Shenzhen 518055 China

**Keywords:** capacity difference mechanisms, conversion‐type transition‐metal sulfides anode, In‐situ magnetometry, Li/Na/K‐ion batteries, spin‐polarized electrons

## Abstract

Conversion‐type transition‐metal sulfides (CT‐TMSs) have been extensively studied as the anode of Li/Na/K‐ion batteries due to their high theoretical capacity. An issue with the use of the material in the battery is that a large capacity difference is commonly observed. However, the underlying mechanism leading to the problem is still unknown. Here, the large capacity difference mechanisms of CT‐TMSs anodes in the Li/Na/K‐ion storage are elucidated, which arises from the difference in conversion degree and size of conversion products. Specifically, the increase in ionic radius will cause the increase in insertion‐reaction ion diffuse energy barrier and conversion‐reaction Gibbs free energies of phase transformation to decrease reaction kinetics, which causes a decrease in conversion degree and an increase in size of conversion products, thus leading to reduction in capacity. The increase in size and the decrease in the amount of conversion products inevitably reduce the amount of spin‐polarized electrons injection into Fe and corresponding ions storage amount into sulfides during the ion‐electron decoupling storage, thus reducing the capacity. The research clarifies the capacity difference mechanisms of CT‐TMSs anodes in Li/Na/K storage, providing valuable insights for designing Li/Na/K storage high‐capacity anodes.

## Graphical abstract

The work elucidates the mechanisms leading to capacity difference of conversion‐type transition‐metal sulfides anodes in Li/Na/K storage, which arise from the differences in ion diffuse energy barrier during the insertion reaction, Gibbs free energies of phase transformation during the conversion reaction, and injection amount of spin‐polarized electrons into Fe during the ion‐electron decoupling storage.

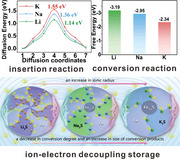

## 1 Introduction

The development of high‐energy‐density lithium‐ion batteries (LIBs), sodium‐ion batteries (SIBs), and potassium‐ion batteries (PIBs) is highly dependent on the capacity of anode materials, in which a higher anode capacity can lead to a higher energy density.^[^ ^1^, ^2^, ^3^, ^4^, ^5^ ^]^ However, the reversible capacities of the dominated commercial graphite and hard/soft carbon anode materials are low in Li/Na/K storage, basically ranging from 200–360 mAh g^−1^, which restricts the further enhancement of energy densities.^[^ ^6^, ^7^, ^8^, ^9^ ^]^ Besides, the dominant commercial graphite anodes commonly used in LIBs are unsuitable for SIBs arising from thermodynamic instability of the binary Na‐intercalated graphite compounds,^[^ ^10^ ^]^ and they displays a low capacity (279 mAh g^−1^) in PIBs.^[^ ^11^ ^]^ Consequently, the development of a universal high‐capacity anode for LIBs, SIBs, and PIBs is of paramount importance.

Conversion‐type transition‐metal sulfides (CT‐TMSs) as LIBs, SIBs, and PIBs universal anodes have become focal points in research due to their uniformly high theoretical capacity in Li/Na/K storage, which are ascribed to their similar conversion mechanisms (M^n+^S + ne^−^ + nLi^+^ → M^0^ + N_2_S, M: transition metals; N: Li, Na or K). Examples include 5d‐TMSs such as tungsten sulfide (432 mAh g^−1^ for WS_2_),^[^ ^12^ ^]^ 4d‐TMSs such as molybdenum sulfide (670 mAh g^−1^ for MoS_2_),^[^ ^1^ ^]^ 3d‐TMSs such as iron sulfides (576 mAh g^−1^ for Fe_1‐x_S;^[^ ^13^ ^]^ 609 mAh g^−1^ for FeS;^[^ ^14^ ^]^ 894 mAh g^−1^ for FeS_2_^[^ ^15^ ^]^), cobalt sulfides (544 mAh g^−1^ for CoS;^[^ ^16^ ^]^ 589 mAh g^−1^ for CoS;^[^ ^17^ ^]^ 870 mAh g^−1^ for CoS_2_^[^ ^18^ ^]^), and nickel sulfides (445 mAh g^−1^ for Ni_3_S_2_; 589 mAh g^−1^ for NiS; 870 mAh g^−1^ for NiS_2_).^[^ ^19^ ^]^ Despite the theoretically identical capacities of CT‐TMSs in Li/Na/K storage, substantial differences in practical electrochemical performance are observed under the same current density. For example, the interoverlapped nanostructure of monolayer WS_2_ and C obtained a high capacity of 1371.2 mAh g^−1^ in LIBs,^[^ ^12^ ^]^ which is ≈2.1 and 3.1 times those achieved in SIBs (661.8 mAh g^−1^)^[^ ^20^ ^]^ and PIBs (449.5 mAh g^−1^),^[^ ^21^ ^]^ respectively. Single‐layered MoS_2_/N,O codoped carbon nanocomposites showed a high capacity of 1520.1 mAh g^−1^ in LIBs, which is about 1.8 and 2.5 times those achieved in SIBs (866.2 mAh g^−1^) and PIBs (620.9 mAh g^−1^), respectively.^[^ ^1^ ^]^ The yolk‐shell Fe_1‐x_S@C composite delivered a high capacity of ≈1300 mAh g^−1^ in LIBs, which is about 2.0 and 2.6 times those obtained in SIBs (≈654 mAh g^−1^) and PIBs (≈505 mAh g^−1^), respectively.^[^ ^15^ ^]^ The hollow CoS/MXene composite nanobox achieved a high capacity of 1128.6 mAh g^−1^ in LIBs, which is ≈1.5 and 3.7 times those obtained in SIBs (734.5 mAh g^−1^) and PIBs (309.0 mAh g^−1^), respectively.^[^ ^22^ ^]^ Clarifying the mechanisms behind these substantial capacity differences is crucial to uncovering the underlying processes of Li/Na/K storage and identifying the key factors governing ion storage capacity. This understanding is essential for developing high‐capacity CT‐TMSs anodes for LIBs, SIBs, and PIBs, yet remains unexplored to date.

Herein, we thoroughly elucidate the mechanisms underlying the capacity differences of CT‐TMSs anodes in Li/Na/K storage, using Fe_1‐x_S as a representative example due to its abundant availability and straightforward preparation process. More crucially, the conversion product, 3d transition metal Fe, undergoes significant magnetic changes when it gains or loses spin‐polarized electrons, making it particularly suitable for in‐situ magnetometry characterizations. By integrating in‐situ magnetometry, density functional theory (DFT) calculations, transmission electron microscopy (TEM), and X‐ray diffraction (XRD) techniques, we discovered that as LIB/SIB/PIB anode the Fe_1‐x_S experiences three distinct processes, namely insertion, conversion, and ion‐electron decoupling. The capacity differences in Fe_1‐x_S across Li/Na/K storage arise from variations in the ion diffusion energy barrier during the insertion reaction, Gibbs free energies of phase transformation during the conversion reaction, and the quantity of spin‐polarized electrons injected into Fe during space charge formation. Specifically, the increase in ionic radius leads to a higher ion diffusion energy barrier (insertion reaction) and Gibbs free energies of phase transformation (conversion reaction), thereby reducing reaction kinetics. This reduction in reaction kinetics results in a lower degree of conversion and larger conversion product sizes, ultimately causing a reduction in capacity. Additionally, the increase in size and the decrease in the quantity of conversion products inevitably reduce the number of spin‐polarized electrons injected into Fe and the corresponding ion storage capacity in sulfides during ion‐electron decoupling storage, further diminishing the overall capacity. Our research elucidates the mechanisms behind the capacity differences of CT‐TMSs anodes in Li/Na/K storage, providing valuable insights for designing high‐capacity anodes for Li/Na/K‐ion batteries.

## 2 Results and Discussion

### 2.1 Material Fabrication, Capacity Testing, and Ion Storage Mechanisms Investigation

**Figure 1:**
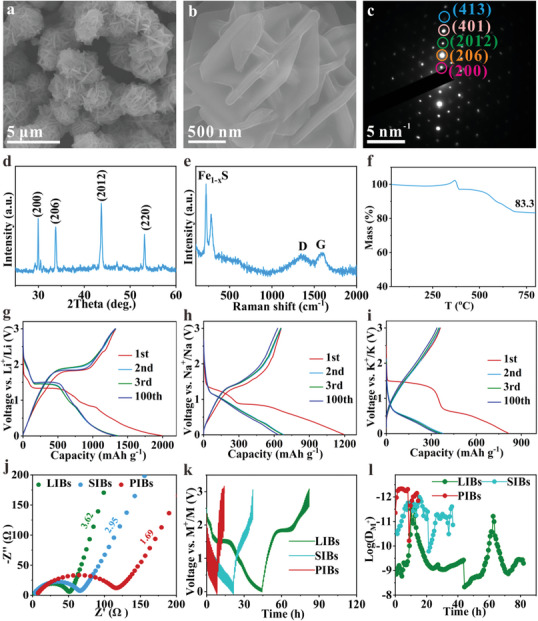
Structural and compositional characterizations of Fe_1‐x_S/C composite: a,b) SEM images, c) SAED pattern, d) XRD pattern, e) Raman spectrum, and f) TGA curve. Electrochemical performance testing: g‐i) Charge/discharge curves in LIBs (g), SIBs (h), and PIBs (i), j) EIS, k,l) GITT curves (k) and the corresponding ion diffusion coefficient (l) of Fe_1‐x_S/C electrodes.

The large volume change during ions process would unavoidably cause structural pulverization of Fe_1‐x_S, which can adversely affect on the clarification of capacity difference mechanisms. Whether academic researches or industrial production, anode materials with large volume expansion are often combined with carbon materials after nanosizing to ensure their structural stability during ion storage.^[^ ^23^ ^]^ Furthermore, to align with industrial production processes, composite anode materials should be designed with a nano‐micro structure, integrating nano‐sized materials into larger micro‐scale sizes. This design approach ensures that the mechanisms revealed are more applicable to providing theoretical guidance for the practical production of CT‐TMSs. Additionally, to better observe the conversion degree between Fe_1‐x_S and ions in different positions of the composites using TEM, a micrometer‐scale structure woven with nanosheets (a plane size in micro and a thickness less than 100 nm) should be a ideal choice. Consequently, we synthesized Fe_1‐x_S/C nanosheets interwoven structure (**Figure** **1**a,b; Figure S1, Supporting Information) with single‐crystal characteristic representing diffraction spots in the selected area electron diffraction (SAED) pattern (Figure 1c). The XRD pattern (Figure 1d) exhibits diffraction peaks corresponding to the (200), (206), (2012), and (220) crystal planes of Fe_1‐x_S (PDF#29‐0725), confirming the formation of Fe_1‐x_S (x value would be determined as 0.1 below). This is further corroborated by the Raman spectrum (Figure 1e), which displays two peaks at 220 cm^−1^ (symmetric stretching) and 280 cm^−1^ (asymmetric stretching) attributed to Fe_1‐x_S,^[^ ^24^ ^]^ as well as two peaks at 1357 cm^−1^ (D peak) and 1590 cm^−1^ (G peak) from carbon materials, confirming the co‐existence of Fe_1‐x_S and carbon. The X‐ray photoelectron spectroscopy (XPS) survey spectrum (Figure S2 and Table S1, Supporting Information) indicates an atomic ratio of 0.9:1 for Fe and S (x = 0.1). In order to further determine the content of each element, elemental analysis tests were performed. The result (Table S2, Supporting Information) shows that the content of Fe is about 58.268 wt.% similar with the result of inductively coupled plasma‐atomic emission spectrometry, and further demonstrates an atomic ratio of 0.9:1 for Fe and S. Fe_1‐x_S can be oxidized into Fe_2_O_3_ in air, resulting in a weight reduction of 12.6 wt.%, corresponding to a final mass fraction of 83.3 wt.% (Figure 1f), which equates to 95.3 wt.% of Fe_1‐x_S in the composite, suggesting a carbon content of 4.7 wt.%. This low carbon content significantly minimizes the impact of carbon on the capacity of the composite electrode.

To clarify the specific capacity difference mechanisms of Fe_1‐x_S in Li/Na/K storage, the corresponding charge/discharge curves in the first three cycles were tested. The Fe_1‐x_S/C shows the first charge/discharge specific capacities of 1330.6/1992.4 mAh g^−1^ in LIBs (Figure 1g), 660.2/1192.5 mAh g^−1^ in SIBs (Figure 1h), and 360.3/814.6 mAh g^−1^ in PIBs (Figure 1i), corresponding to the first Coulombic efficiency of 66.8, 55.4, and 44.2%, respectively. The main reasons that the initial discharge capacity of Fe_1‐x_S/C exceeds its theoretical capacity are ascribed to the formation of the irreversible solid electrolyte interphase (SEI) layers due to electrolyte decomposition^[^ ^20^ ^]^ and the construction of space charge storage at the interfaces of the formed superparamagnetic Fe and the corresponding sulfides during conversion reaction (discussed in detailed below). Clearly, the first reversible specific capacity obtained in LIBs is the highest, followed by SIBs, and the lowest in PIBs, in which the capacity of in LIBs is 2.0 and 3.7 times the capacity of SIBs and PIBs, respectively. After 100 cycles, the Fe_1‐x_S/C electrodes still deliver high reversible capacities of 1322.7, 631.7, and 340.1 mAh g^−1^ in LIBs, SIBs, and PIBs demonstrating good capacity retentions of 99.4, 95.7, and 94.4%, respectively. The results indicate high electrode stability during ions storage. Electrochemical impedance spectra (EIS) and galvanostatic intermittent titration technique (GITT) curves (Related GITT formula is introduced in experimental section) are tested to illustrate the differences of ion diffusion kinetics in Li/Na/K storage. Obviously, with an increase in ionic radius (1.38 Å for K‐ion; 1.02 Å for Na‐ion; 0.76 Å for Li‐ion) the charge transfer resistance (R_ct_, diameter of depressed semicircle in high‐frequency zone) represents an increased trend and the slope of the oblique line (Warburg impedance of ion diffusion in low‐frequency zone) shows a reduced trend (Figure 1j). The GITT (Figure 1k) and their corresponding Li/Na/K‐ion diffusion coefficient (D_Li+_, D_Na+_, and D_K+_) curves (Figure 1l) display that the D_Li+_ (3.3 × 10^−12^‐5.0 × 10^−9^ cm^2^ s^−1^) of Fe_1‐x_S/C electrodes are higher than D_Na+_ (1.0 × 10^−12^‐1.7 × 10^−10^ cm^2^ s^−1^) and D_K+_ (4.6 × 10^−13^‐6.2 × 10^−11^ cm^2^ s^−1^). The EIS and GITT data indicate the increase of ion diffuse resistance with an increase in ionic radius, which can cause the large capacity difference in Li/Na/K storage. However, this is only a basic explanation on the capacity differences in Li/Na/K storage from the perspective of ion diffusion kinetics, lacks deeper analysis. To thoroughly analyze the substantial capacity differences in Li/Na/K storage, it is necessary to investigate the corresponding ion storage mechanisms of Fe_1‐x_S. For this purpose, ex‐situ XRD, ex‐situ TEM, in‐situ magnetometry, and DFT calculations were performed sequentially. First, ion storage mechanisms of Fe_1‐x_S/C electrodes are probed by ex‐situ XRD. Apparently, discharging to 1.6 (LIBs, Figure S3, Supporting Information), 1.4 (SIBs, Figure S4, Supporting Information), and 1.3 (PIBs, Figure S5, Supporting Information) V, the diffraction peaks of Fe_1‐x_S shift toward a lower angle ascribed to the insertion reaction. In LIBs (Figure S3, Supporting Information), continuing to discharge to 0.01 V, the diffraction peaks of Fe_1‐x_S in LIBs disappear completely along with the appearance of diffraction peak of (200) crystal plane of Li_2_S (PDF#23‐0369) at ≈31.3° due to the conversion reaction. In SIBs (Figure S4, Supporting Information), a new peak appears at ≈34.7  ascribed to (611) crystal plane of Na_2_S (PDF# 47–0178) along with co‐existence of the diffraction peaks of Fe_1‐x_S when discharge to 0.01 V, indicating that the Fe_1‐x_S undergoes an incomplete conversion reaction during Na storage. Similar results also appear in PIBs, a new broad peak appears at ≈34.3° ascribed to (220) crystal plane of K_2_S (PDF# 47–1702) along with co‐existence of the diffraction peaks of Fe_1‐x_S when discharge to 0.01 V (Figure S5, Supporting Information), indicating that the Fe_1‐x_S undergoes an incomplete conversion reaction during K storage.^[^ ^25^ ^]^ After charging to 3 V (Figures S3‐S5, Supporting Information), the diffraction peaks of Fe_1‐x_S only exist, suggesting the high reversibility of conversion reaction in Li/Na/K storage. The lack of diffraction peaks of Fe nanocrystals results from its ultrasmall size,^[^ ^26^, ^27^ ^]^ as confirmed in TEM observations below. Consequently, the XRD results demonstrate the insertion and conversion mechanisms of Fe_1‐x_S in ions storage and reveal an incomplete conversion reaction during Na/K storage.

**Figure 2:**
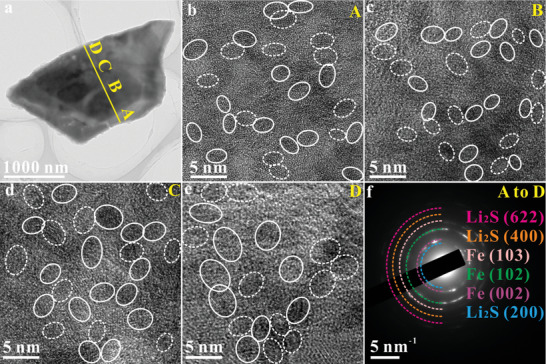
Ex‐situ TEM characterization in LIBs: a–e) TEM images and f) the representative SAED pattern. Images b, c, d, and e are taken from points A, B, C, and D, respectively of image a.

**Figure 3:**
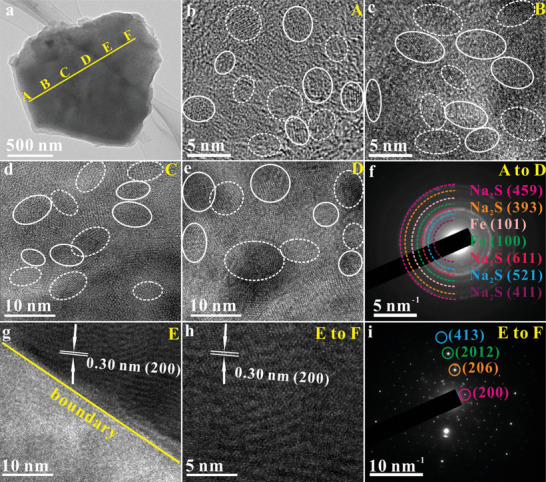
Ex‐situ TEM characterization in SIBs: a–e) TEM images and f) the representative SAED pattern from point A‐D. Images b, c, d, and e are taken from points A, B, C, and D, respectively of image a. g) TEM image taken from point E. h) TEM image and i) the corresponding SAED pattern taken from point E to F.

**Figure 4:**
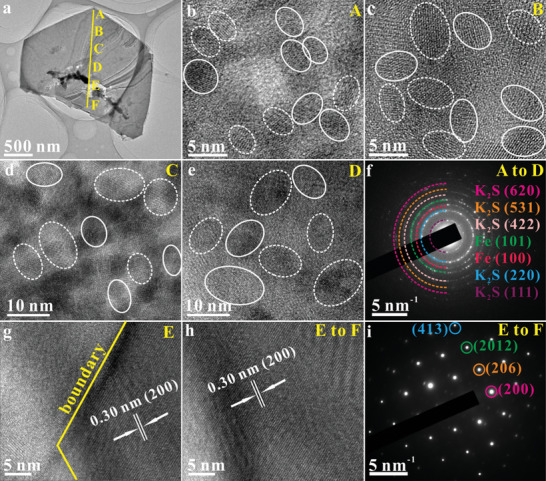
Ex‐situ TEM characterization in PIBs: a–e) TEM images and f) the representative SAED pattern from point A‐D. Images b, c, d, and e are taken from points A, B, C, and D, respectively of image a. g) TEM image taken from point E. h) TEM image and i) the corresponding SAED pattern taken from point E to F.

Second, ex‐situ TEM characterizations (**Figures** 2, 3, 4) were performed to further confirm ion storage mechanisms of Fe_1‐x_S after discharging to 0.01 V. To facilitate the observation of the nanosheets’ microstructure at different areas, the overall thickness of the nanosheets was kept below 100 nm. Nevertheless, the size of the interwoven structures is micrometer scale (Figure 1a), comprehensively observing the microstructure of individual nanosheets is challenging. Consequently, the interwoven structures should be disengaged by sonication in ethanol for a specific duration, thus achieving thin nanosheets to be observed. It should be noted that in the following TEM images the crystallized Fe and Li_2_S/Na_2_S/K_2_S nanoparticles are labeled with white solid and dashed circles, respectively. Along the straight yellow line from point A to D in image a (Figure 2a), we captured a series of TEM images and SAED patterns, with representative TEM images and SAED pattern shown in Figure 2b–f. Clearly, across the nanosheet from edge (A) near the carbon layers to nanosheet's center (D), the Fe_1‐x_S is fully transformed into crystalline Fe and Li_2_S nanoparticles possessing the sizes of ≈2.5 nm (Figure 2b, point A), ≈3.0 nm (Figure 2c, point B), ≈3.4 nm (Figure 2d, point C), and 3.8 nm (Figure 2e, point D). The representative SAED pattern (Figure 2f) represents polycrystalline diffraction rings attributed to (002), (102), and (103) of Fe (PDF#34‐0529) and (200), (400), and (622) of Li_2_S (PDF#23‐0369). The results indicate that the Fe_1‐x_S nanosheet undergoes a complete conversion during lithium storage in accordance with the ex‐situ XRD results (Figure S3, Supporting Information).

In SIBs, along the straight yellow line from point A to F in image a (Figure 3a), we took a series of TEM images and SAED patterns, in which typical TEM images and SAED patterns are shown in Figure 3b–i. Clearly, across the nanosheet from point A to D, the Fe_1‐x_S is transformed into crystalline Fe and Na_2_S nanoparticles with the sizes of ≈3.6 nm (Figure 3b, point A), ≈4.9 nm (Figure 3c, point B), ≈6.1 nm (Figure 3d, point C), and 6.9 nm (Figure 3e, point D). The crystallized Fe and Na_2_S nanoparticles are labeled with white solid circles and white dashed circles, respectively. The representative SAED pattern (Figure 3f) shows the polycrystalline diffraction rings ascribed to (100) and (101) crystal planes of Fe (PDF# 34–0529) and (411), (521), (611), (393), and (459) crystal planes of Na_2_S (PDF# 47–0178). However, at point E, a distinct boundary between the conversion‐product Fe/Na_2_S and untransformed single‐crystal Fe_1‐x_S is observed (Figure 3g). From point E to F, pristine single‐crystal Fe_1‐x_S structure remains intact (Figure 3h,i), indicating that the Fe_1‐x_S nanosheet undergoes an incomplete conversion during sodium storage in accordance with the ex‐situ XRD results (Figure S4, Supporting Information).

Similar results also appear in PIBs (Figure 4). Along the straight yellow line from point A to F in image a (Figure 4a), we obtained a series of TEM images and SAED patterns, with representative examples TEM images and SAED patterns shown in Figure 4b–i. Across the nanosheet from point A to D, the Fe_1‐x_S is transformed into crystalline Fe and K_2_S nanoparticles with the sizes of ≈4.2 nm (Figure 4b, point A), ≈5.6 nm (Figure 4c, point B), ≈8.7 nm (Figure 4d, point C), and 10.2 nm (Figure 4e, point D). The crystallized Fe and K_2_S nanoparticles are labeled with white solid circles and white dashed circles, respectively. The representative SAED pattern (Figure 4f) exhibits the polycrystalline diffraction rings arising from (100) and (101) crystal planes of Fe (PDF# 34–0529) and (111), (220), (422), (531), and (620) crystal planes of K_2_S (PDF# 47–1702). However, at point E, an obvious boundary between the conversion‐product Fe/K_2_S and untransformed single‐crystal Fe_1‐x_S is observed (Figure 4g). From point E to F, the pristine single‐crystal Fe_1‐x_S structure remains intact (Figure 4h,i), suggesting that the Fe_1‐x_S nanosheet undergoes an incomplete conversion during potassium storage in accordance with the ex‐situ XRD results (Figure S5, Supporting Information). It should be noted that a combination of TEM observations and ex‐situ XRD patterns reveals that Fe_1‐x_S is ultimately converted to crystalline Fe and Li_2_S/Na_2_S/K_2_S in LIBs, SIBs, and PIBs, respectively, without other intermediate phases.

**Figure 5:**
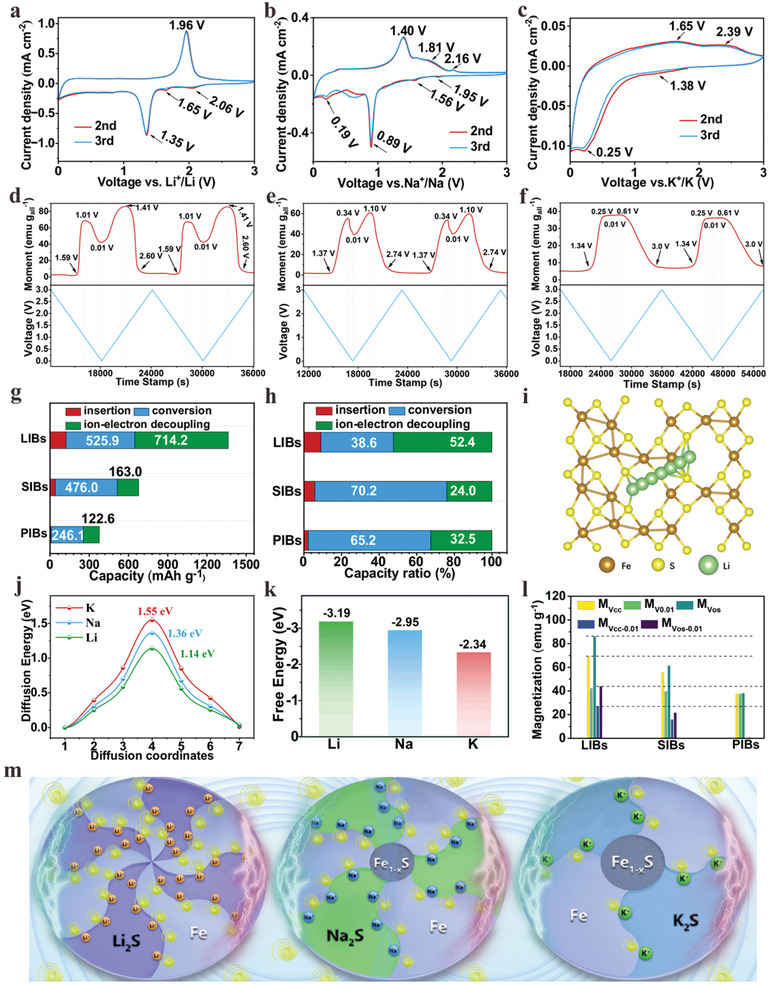
*In‐situ* magnetometry characterizations: a–c) CV curves at 0.5 mV s^−1^ in LIBs a), SIBs b), and PIBs c); d‐f) magnetic response spectra in LIBs d), SIBs e), and PIBs f); g) Specific capacity values and h) capacity ratio in total capacity from ion storage mechanism of insertion, conversion, and ion‐electron decoupling. DFT calculations: i) Top view of configurations of lithium migration paths, j) Ion diffusion energy barrier curves, and k) Gibbs free energy histogram. l) Specific magnetization values from different voltage points. m) Diagrammatic sketch of ion‐electron decoupling in Li/Na/K‐ion storage.

Similarly, in LIBs, SIBs, and PIBs, the size of the conversion products shows an increased trend from nanosheet's edge to its center. The variation in nanocrystals size may be caused by differences in ion reaction kinetics across different regions of nanosheet. The outermost exposed nanosheet edges in the interwoven structure are in greater contact with the electrolyte compared to the root of the nanosheet, leading to quicker and more complete reactions with ions, thereby facilitating the formation of smaller nanocrystals. Differently, the nanocrystal size formed by the conversion reaction has the maximum in PIBs (4.2 nm at edge and 10.2 nm at center), medium in SIBs (3.6 nm at edge and 6.9 nm at center), and minimum in LIBs (2.5 nm at edge and 3.8 nm at center). This can be ascribed to the fastest reaction kinetics between lithium ions and Fe_1‐x_S/C owing to its highest ion diffusion coefficient (Figure 1l), lowest ion diffusion energy barrier in the insertion reaction (as shown in **Figure** **5**), and lowest Gibbs free energy required for the conversion reaction (as illustrated in Figure 5), which result in the formation of the smallest nanocrystals.

The conversion degree of Fe_1‐x_S and the resulting size variation of the formed nanocrystals during the conversion reaction are the primary factors contributing to the difference in capacity of Fe_1‐x_S/C among the LIBs, SIBs, and PIBs. This will be further analyzed by *in‐situ* magnetometry, where the plot of magnetization versus voltage is recorded while testing cyclic voltammetry (CV) curves at 0.5 mV s^−1^ (Figure 5). Given the significant differences in the first Coulombic efficiency (Figure 1g–i) of Fe_1‐x_S in Li/Na/K‐ion batteries, magnetic spectra and charge/discharge curves from the second and third cycles were selected for analysis to eliminate the influence of the irreversible capacity during the first discharge process and to better understand the mechanisms behind the capacity differences.

In Figure 5a for LIBs, during the discharge scan the CV curves exhibit two weak peaks at 2.06 and 1.65 V, and a strong peak at 1.35 V, corresponding to the stepwise insertion and conversion reactions, respectively.^[^ ^28^, ^29^ ^]^ In the charge scan, an oxidation peak at 1.96 V is due to the oxidation of Fe/Li_2_S.^[^ ^28^, ^29^ ^]^ In Figure 5b for SIBs, during the discharge scan the CV curves exhibit four peaks at 1.95, 1.56, 0.89, and 0.19 V attributed to the stepwise insertion (1.95 and 1.56 V) and conversion (0.89 and 0.19 V) reactions.^[^ ^24^ ^]^ In the charge scan, three oxidation peaks at 1.40, 1.81, and 2.16 V are due to the stepwise oxidation of Fe/Na_2_S.^[^ ^24^ ^]^ In Figure 5c for PIBs, during the discharge scan the CV curves exhibit two small peaks at 1.38 and 0.25 V, which correspond to the insertion and conversion reactions, respectively.^[^ ^15^ ^]^ In the charge scan, two oxidation peaks at 1.65 and 2.39 V are due to the stepwise oxidation of Fe/K_2_S.^[^ ^15^ ^]^ The CV curves further prove the insertion and conversion reaction mechanisms of Fe_1‐x_S in Li/Na/K storage. *In‐situ* magnetometry (Figure 5d) of LIBs displays that the magnetization presents an almost constant value from initial voltage to conversion‐reaction beginning voltage (Vcb, ≈1.59 V), suggesting that electrode magnetization is not influenced by the insertion reaction. From Vcb to conversion‐reaction cutoff voltage (Vcc, ≈1.01 V), the magnetization quickly goes up to reach a maximum at Vcc resulting from the appearance of Fe nanoparticles formed through conversion reaction. Going on discharging to 0.01 V, the quick reduction of magnetization is represented, indicating that spin‐polarized electrons are injected and stored into spin‐down bands of Fe according to the net magnetization equation^[^ ^30^ ^]^ (related information can be found in experimental section). Meanwhile, Li ions can be saved in Li_2_S to maintain charge conservation, which realizes ion‐electron decoupling and obeys Maier's theoretical model of space charge storage.^[^ ^31^ ^]^ The formation of space charge zone can obviously enhance ions storage sites, thereby enhancing the capacity.^[^ ^30^, ^31^ ^]^ This further explains the reason that the initial discharge capacity exceeds the theoretical capacity. From 0.01 to oxidation‐reaction starting voltage (Vos, ≈1.41 V), the magnetization goes up ascribed to the electrons escape from Fe. The magnetization reduces quickly from Vos to ≈2.60 V and retains an almost constant value from ≈2.60 to 3 V attributed to the oxidation of Fe into Fe_1‐x_S (Figure S3, Supporting Information). The two cycles show a basically consistent magnetization evolution, indicating a high reversibility of magnetic responses with respect to voltage. The *in‐situ* magnetometry of SIBs (Figure 5e) and PIBs (Figure 5f) exhibits similar change trends with LIBs, indicating their similar ions storage mechanisms. The difference lies in the magnetization values achieved at key voltage nodes and their changes during ion‐electron decoupling storage. These changes are related to the degree of the conversion reaction and the size of the corresponding formed nanoparticles (discussed below). In brief, the *in‐situ* magnetometry proves that as LIBs/SIBs/PIBs anode Fe_1‐x_S experiences three distinct processes, namely ion‐insertion, phase‐conversion, charge‐decoupling processes.

To demonstrate the universality of the three ion storage mechanisms in other CT‐TMSs, we synthesized Ni_3_S_2_/C and MoS_2_/C composites and tested their CV curves in LIBs, SIBs, and PIBs, which are shown in Figures S6 and S7 (Supporting Information), respectively. Similar with Fe_1‐x_S, the Ni_3_S_2_ and MoS_2_ also undergo insertion and conversion reaction mechanisms during Li/Na/K‐ion storage (Figures S6d‐f and S7d‐f, Supporting Information). Besides, from V_cc_ to 0.01 V and from 0.01 V to Vos, it is observed that the CV curves show a rectangle‐like shape without redox peaks, indicating that the corresponding capacity arises from the pseudo‐capacitance contribution. We take LIBs as an example and test the CV curves from 0.1 to 1 mV s^−1^ in the voltage range of 0.01‐1 V (Figures S6g and S7g, Supporting Information). The calculated b values are ≈1 (Figures S6h and S7h, Supporting Information), indicating complete pseudo capacity contribution in this voltage range (Figures S6i and S7i, Supporting Information), which is consistent with the behavior of space charge storage mechanism.^[^ ^30^ ^]^ The above results confirm the universality of the insertion, conversion, and space charge storage mechanisms in the CT‐TMSs.

The corresponding capacity and capacity ratio at the three processes in LIBs, SIBs, and PIBs can be determined by analyzing the key voltage nodes obtained from magnetometry, in combination with the corresponding charge‐discharge curves (Figure S8, Supporting Information). It should be noted that the carbon coating layers can also store Li/Na/K‐ions. The capacity of carbon can be considered as ≈360, 320, and 225 mAh g^−1^ as LIBs,^[^ ^8^ ^]^ SIBs,^[^ ^7^ ^]^ and PIBs^[^ ^32^ ^]^ anodes, respectively. Based on its mass fraction (Figure 1f), its contribution capacities in LIBs, SIBs, and PIBs are ≈16.9, 15.0, and 10.6 mAh g^−1^, respectively, which are low contribution ratio relative to total capacity. Therefore, the capacity contribution of carbon will not be considered in the following discussions. The capacity obtained from initial voltage to Vcb corresponds to the insertion‐reaction contributed capacity. The capacity obtained from Vcb to Vcc represents conversion‐reaction contributed capacity. The capacity obtained from Vcc to 0.01 V is attributed to ion‐electron decoupling storage.

Based on this principle, the capacities and corresponding ratio for the three processes in LIBs, SIBs, and PIBs are summarized in Tables S3–S5 (Supporting Information), and displayed in Figure 5g,h, respectively. Obviously, the corresponding capacities for insertion, conversion, and ion‐electron decoupling storage are the highest in LIBs, followed by SIBs, and with PIBs showing the lowest capacities. The difference in insertion‐reaction capacity is attributed to the difference in ion diffusion energy barriers. As shown by the DFT results (Figure 5i,j; Figure S9, Supporting Information), the diffusion energy barrier for Li^+^ is lower than that for Na^+^, and both are lower than that of K^+^. A lower ion diffusion energy barrier can allow a faster ion diffusion rate based on Arrhenius‐like equation (introduced in detail in experimental section), thus benefiting for more ion storage and obtaining a higher specific capacity. The calculated values are high relative to other systems,^[^ ^1^, ^12^, ^20^ ^]^ which may result from the difference of structure models, diffusion paths, and/or calculation parameter setting, etc. For Fe_1‐x_S materials, the contribution capacity ratios of the insertion reaction are the lowest (9.0% in LIBs, Table S3, Supporting Information; 5.8% in SIBs, Table S4, Supporting Information; 2.3% in PIBs, Table S5, Supporting Information) among all the three charge/discharge processes. Consequently, the effect of high ion diffusion energy barriers during the insertion reaction on the whole charge/discharge process is small. It should be noted that in calculating the ion diffusion energy barriers, although there are multiple diffusion paths in the constructed structure model (Figure S10, Supporting Information), in our work the purpose of calculating the diffusion energy barrier is to compare the differences in diffusion energy barriers of different ions under the same diffusion path, not to optimize which diffusion path is better. Therefore, we only chose one diffusion path for calculation. The difference in conversion‐reaction capacity is attributed to the difference in phase‐transformation driving force. As shown by the DFT results (Figure 5k, and the corresponding configurations are shown in Figure S11, Supporting Information), the Gibbs free energy change required for conversion reaction is the lowest in LIBs and the highest in PIBs. A lower Gibbs free energy change facilitates the progress of conversion reaction, thereby improving the conversion‐reaction capacity.

Decoupled ions and electrons only exist at the interfaces between the two phases, with a depth of approximately a few atomic layers.^[^ ^31^ ^]^ It is evident that a smaller nanocrystal size and a greater number of nanocrystals lead to more interfaces, which can store more electrons and ions. As confirmed by TEM (Figures 2, 3, 4), the two phases (Fe and Li_2_S) in LIBs exhibit the smallest size and greatest quantity, whereas the two phases (Fe and K_2_S) in PIBs have the largest size and the smallest quantity. Consequently, Fe in LIBs can accommodate the most spin‐polarized electrons, and Li_2_S can store the most Li⁺ ions. The increased injection of spin‐polarized electrons into Fe, coupled with the increased ion storage in sulfides, results in a greater change in magnetization from Vcc to 0.01 V and from 0.01 to Vos, denoted as M_Vcc‐0.01_ and M_Vos‐0.01_, respectively. As depicted in Figure 5l (Table S6, Supporting Information), the M_Vcc‐0.01_ and M_Vos‐0.01_ in LIBs are the highest, followed by SIBs, with PIBs showing the lowest values. The results indicate that ion‐electron decoupling storage capacity is positively correlated with magnetization change (M_Vcc‐0.01_ and M_Vos‐0.01_). In LIBs, SIBs, and PIBs, the total capacity contribution of insertion and conversion are 648.4, 515.1, and 254.6 mAh g^−1^, respectively (Figure 5g; Figure S8, Supporting Information). The 648.4 mAh g^−1^ in LIBs exceeds the theoretical capacity (586 mAh g^−1^, calculated based on insertion and conversion reactions and its corresponding calculated details are introduced in experimental section) of Fe_1‐x_S, which is ascribed to the occurrence of ion‐electron decoupling storage during the conversion reaction. As observed in Figure 5d and displayed in Figure 5l, the M_Vos_ is higher than that M_Vcc_, which suggests that, from the Vcb to Vcc, electrons have injected into the formed Fe to reduce the electrode magnetization, thus offsetting part of the increased magnetization values.

The phenomenon also appears in SIBs (Figure 5e) and PIBs (Figure 5f). This result indicates that the contributed capacity derived from conversion reaction derived above is slightly higher than the actual value. However, we are currently unable to separate the capacity stored by ion‐electron decoupling from the conversion‐reaction process. Therefore, for simplicity, the contribution of ion‐electron decoupling storage to capacity would be not considered during the conversion process in the following discussions. The insertion and conversion capacities in SIBs (515.1 mAh g^−1^) and PIBs (254.6 mAh g^−1^) account for 79.4 and 39.3%, respectively of the obtained capacity (648.4 mAh g^−1^) in LIBs, which can be approximately considered sodification and potassiation degree of Fe_1‐x_S. The maximum electrode magnetization is caused by the formed Fe during the conversion reaction and the conversion is complete in LIBs. If the influence of Fe size on the magnetization value is excluded, the conversion degrees of Fe_1‐x_S in SIBs and PIBs can be determined by the ratio of M_Vos_ in SIBs and PIBs to M_Vos_ in LIBs, which are 71.0 and 44.2% (Table S6, Supporting Information), respectively and further indicate the incomplete conversion of Fe_1‐x_S in SIBs and PIBs. Additionally, the ratios of ion‐electron decoupling storage to total capacity (Figure 5h) are 52.4% (Li), 24.0% (Na), and 32.5% (K), highlighting the significant contribution of ion‐electron decoupling storage to overall capacity. In particular, in LIBs, the ion‐electron decoupling storage contribution exceeds the combined contribution of insertion and conversion. This finding suggests that enhancing the capacity contribution of ion‐electron decoupling storage is an effective strategy for improving the overall capacity of CT‐TMSs in SIBs and PIBs.

Based on the above results, Fe_1‐x_S exhibits the insertion, conversion, and ion‐electron decoupling storage mechanisms in Li/Na/K storage and the corresponding capacity difference mechanisms in Li/Na/K storage arise from the differences in ion diffusion energy barrier during the insertion reaction, Gibbs free energies of phase transformation during the conversion reaction, and injection amount of spin‐polarized electrons into Fe during the ion‐electron decoupling storage related to conversion degree and size of conversion products (Figure 5m). A smaller ion diffuse energy barrier in the insertion reaction, a lower Gibbs free energies of phase transformation during conversion reaction, and a more spin‐polarized electrons injection into Fe (Figure 5m) during the ion‐electron decoupling storage are all favorable for enhanced ion storage. The significance of our work lies in providing theoretical guidance for researchers studying CT‐TMSs or other CT‐TM compounds. To achieve higher reversible capacities in synthesized CT‐TMSs or other CT‐TM compounds, it is crucial to focus on their conversion degree and the size of the conversion products, which are often linked to the material structure and the conductive network of the entire electrode. A material structure and electrode conductive network that is more conducive to charge transport will be more beneficial in improving the conversion degree and reducing the size of the conversion products, thereby increasing their reversible capacities.

## 3 Conclusions

In conclusions, we comprehensively elucidate the mechanisms leading to the capacity difference of CT‐TMSs anodes in Li/Na/K storage by selecting Fe_1‐x_S as a representative material and employing *in‐situ* magnetometry, DFT calculation, TEM, and XRD techniques. Our findings reveal that Fe_1‐x_S electrodes undergo three processes in Li/Na/K storage: insertion, conversion, and ion‐electron decoupling storage. The capacity differences of Fe_1‐x_S in Li/Na/K storage arise from variations in conversion degree and conversion products size. Specifically, a smaller ionic radius favors for the decrease of ion diffuse energy barrier during the insertion reaction and Gibbs free energies of phase transformation during the conversion reaction, which can increase reaction kinetics to ensure more sufficient conversion and reduce the size of conversion products, thus increasing the capacity during insertion and conversion reaction. Besides, more sufficient conversion can increase the corresponding amount of conversion products. The decrease in size and the increase in the amount of conversion products can raise the amount of spin‐polarized electrons injection into Fe and corresponding ions storage amount into sulfides, thus improving the capacity during the ion‐electron decoupling storage. Our research clarifies the capacity difference mechanisms of CT‐TMSs anodes in Li/Na/K storage, providing valuable insights for designing high‐capacity anodes for Li/Na/K‐ion batteries.

## Conflict of Interest

The authors declare no conflict of interest.

## Author Contributions

K.Z. and Y.M. contributed equally to this work. K.Z. and Y.M. performed investigation, data curation, and writing‐original draft preparation. M.H. performed conceptualization, methodology, supervision, writing‐review & editing, and funding acquisition. J.L., Z.Z., H.H., Y.C., and F.Y. performed investigation. W.L. and L.W. performed methodology. L.Z. and T.Z. performed writing‐review & editing, funding acquisition.

## Supporting information



Supporting Information

## Data Availability

The data that support the findings of this study are available from the corresponding author upon reasonable request.
