# Supplementary material for: Investigations of Mechanisms Leading to Capacity Differences in Li/Na/K‐Ion Batteries with Conversion‐Type Transition‐Metal Sulfides Anodes
Source: Adv Sci (Weinh). 2024 Nov 4;11(48):2410653. doi: 10.1002/advs.202410653 (PMC11672257; doi:10.1002/advs.202410653)
Supplement: Supplementary file 1 — Supporting Information [file ADVS-11-2410653-s001.pdf]

ADVANCED SCIENCE
Open Access

Supporting Information

for *Adv. Sci.*, DOI 10.1002/advs.202410653

Investigations of Mechanisms Leading to Capacity Differences in Li/Na/K-Ion Batteries with Conversion-Type Transition-Metal Sulfides Anodes

Kunxiong Zheng, Yongbiao Mu, Meisheng Han, Jie Liu, Zhiyu Zou, Hengyuan Hu, Youqi Chu, Fenghua Yu, Wenjia Li, Lei Wei, Lin Zeng* and Tianshou Zhao**

Supporting information for

Investigations of Mechanisms Leading to Capacity Differences in Li/Na/K-Ion Batteries with Conversion-Type Transition-Metal Sulfides Anodes

Kunxiong Zheng,[#1,2] Yongbiao Mu,[#1,2] Meisheng Han,[*1,2] Jie Liu,[1,2] Zhiyu Zou,[1,2] Hengyuan Hu, Youqi Chu,[1,2] Fenghua Yu,[1,2] Wenjia Li,[1,2] Lei Wei,[1,2] Lin Zeng,[*1,2] and Tianshou Zhao[*1,2]

[1]Shenzhen Key Laboratory of Advanced Energy Storage, Department of Mechanical and Energy Engineering, Southern University of Science and Technology, Shenzhen 518055, China

[2]SUSTech Energy Institute for Carbon Neutrality, Southern University of Science and Technology, Shenzhen 518055, China

*Corresponding Authors. E-mail address: hanms@sustech.edu.cn (M. Han); zengl3@sustech.edu.cn (L. Zeng); zhaots@sustech.edu.cn (T. Zhao)

#These authors contributed to this work equally.

Experimental Section

Material characterization:

The morphology was characterized by SEM (Hitachi SU-8230). TEM was performed using a Talos instrument with an acceleration voltage of 300 kV. XPS (Thermo Fisher ESCALAB Xi^{+}) was acquired with Al Kα (hυ = 1486.8 eV) as the excitation source. Raman spectrum was tested on a Horiba Labram HR Evolution using a 532 nm laser. XRD (D/max-2500/PC, Rigaku) was used to test the crystal structure. Elemental analyzer (Perkinelmrr 2400 Series II) and Inductively coupled plasma-atomic emission spectrometry (ICP-AES, iCAP7400 Duo MFC) were used to determine the composition of each element. TGA was tested in oxygen atmosphere using the Pyris I, PerkinElmer instrument over the temperature range of room temperature to 800 °C with a heating rate of 10 °C min^{-1}.

In-situ Magnetometry Experiment

This test has received the support of Professor Qiang Li from Qingdao University, and We would like to express our gratitude to Professor Qiang Li.

The magnetometry test devices were assembled using flexible packaging batteries in an argon-filled glovebox at room temperature. Operando magnetometry experiments were conducted using a Quantum Design superconducting quantum interference device at 25 °C. Magnetic measurements were consistently performed at an applied magnetic field of 3 T, oriented parallel to the surface of the copper foil. Simultaneously, operando magnetic measurements were performed in conjunction with electrochemical workstation in the form of testing CV curves at 0.5 mV s^{-1}. To extract the relevant magnetic data, linear magnetic background signals originating from other components of the cell assembly were meticulously subtracted from the total magnetic moment.

Electrochemical measurements:

Half-cell assessment: To assess the electrochemical behaviour of all samples, CR2032-type coin cells were assembled. The working electrodes were composed

of active materials, acetylene black, and polyvinylidene fluoride at a mass ratio of 8:1:1. The obtained electrodes were dried under vacuum at 90 °C for at least 12 h. For LIBs, lithium metal piece was served as the counter/reference electrode, Celgard 2400 membrane was used as separator absorbing electrolyte (1 M $LiPF_6$ in a mixture of ethylene carbonate (EC)/diethylene carbonate (DEC)/dimethyl carbonate (DMC) at a volume ratio of 1:1:1 with 5 wt% fluoroethylene carbonate (FEC)). For SIBs, sodium metal piece was served as the counter/reference electrode, using Whatman glass fiber as separator absorbing electrolyte (1 M $NaPF_6$ in a mixture of EC and DEC 1:1 (vol%) with 5 wt% FEC). For PIBs, potassium metal piece was served as the counter/reference electrode using Whatman glass fiber as separator absorbing electrolyte (1.0 M potassium bis(fluorosulfonyl)imide dissolved in the mixture of EC and DEC with a volume ratio of 1:1 with 5 wt% FEC). The mass loading of active materials is around 1.20 mg cm^{-2}. The electrochemical performance was evaluated using a Neware battery test system (Shenzhen, China) at temperatures of 25 ^{o}C. CV curves at scanning rates of 0.1-20.0 mV s^{-1} and EIS with frequency ranges of 10^5 to 10^{-2} Hz were conducted using a CHI 760D electrochemical workstation (Shanghai CH Instruments Co., China).

Calculation of theoretical capacity for $Fe_{1-x}S$:

For $Fe_{1-x}S$, one $Fe_{1-x}S$ can store 2(1-x) A^+ (A: Li, Na, and K), resulting in the overall ion storage reaction:

$$Fe_{1-x}S + (2\text{-}2x)A^+ + (2\text{-}2x)e^- \rightarrow (1\text{-}x)Fe + A_{2-2x}S$$

The theoretical specific capacity (C_g) is calculated using the formula:

$$C_g = \frac{n \times F}{M} \div 3.6$$

Number of Electrons Transferred n = (2-2x) moles of electrons per mole of $Fe_{1-x}S$.

Faraday's Constant F = 96485 C/mol.

Molar Mass of $Fe_{1-x}S$ $M = (1-x) \times M_{Fe} + M_S$ g/mol.

When x = 0.1， n = 1.8. Substituting the values:

$$C_g = \frac{1.8 \times 96485}{82.265} \div 3.6 \approx 586\ \text{mAh/g}$$

We have derived the atomic ratio of Fe and S from XPS survey spectra (Table S1), elemental analysis tests and ICP-AES (Table S2) to be about 0.9, which corresponds to x = 0.1 and a theoretical capacity of about 586 mAh/g.

"Arrhenius-like equations" for ion diffusion D:

$$D = D_0 exp\left(-\frac{E_a}{k_B T}\right)$$

Where:

D: Diffusion coefficient (unit: $m^2 \cdot s^{-1}$).

D_0: Pre-exponential factor (unit: $m^2 \cdot s^{-1}$), related to the material being diffused.

E_a : Activation energy (unit: $J \cdot mol^{-1}$) can represent diffusion energy barrier during ion diffusion process.

k_B: Gas constant (8.314 $J \cdot mol^{-1} \cdot K^{-1}$).

T : Absolute temperature (unit: K).

This equation describes how ion diffusion rate (which impacts ion transport and eventually capacity) increases as the energy barrier decreases. Higher diffusion rate contributes to higher specific capacity, especially at higher charge/discharge rates. Specifically, a smaller ionic radius facilitates a decrease in the ion diffusion energy barrier during the insertion reaction, thereby increasing the capacity during insertion reaction. This is consistent with the results of our performed DFT simulations (Figure 5j).

Calculation of ion diffusion coefficient based on the GITT measurement:

The formula for calculating D is as follows:

$$D = \frac{4}{\pi\tau}\left(\frac{m_B V_M}{M_B S}\right)^2 \left(\frac{\Delta E_S}{\Delta E_\tau}\right)$$

where M_B, m_B, and V_M are molar mass, mass, and molar volume of the active material, respectively. S, τ, ΔE_S, and ΔE_τ represent electrode area, constant current time, and voltage change, as well as total voltage change, respectively. The values of τ, ΔE_S, and ΔE_τ are determined from the GITT curves.

Net magnetization equation:

Specifically, $M = (N\uparrow - N\downarrow) * \mu B$ ($N\uparrow$-the number of spin-up electrons; $N\downarrow$- the number of spin-down electrons; μB-Bohr magneton). The decrease of magnetization is ascribed to that the spin-polarized electrons enter into spin-down d bands. This is because the spin-up d bands of Fe are filled much more than its spin-down d bands. To maintain charge conservation, ions can be stored in the corresponding formed sulfides, which realizes ion-electron decoupling and obeys Maier's theoretical model of space charge storage.

DFT calculation:

All the DFT calculations were conducted based on the Vienna Ab-inito Simulation Package[1-2]. The DFT-D3 method was used to describe van der Waals (vdW) interactions.[3] The exchange-correlation effects were described by the Perdew-Burke-Ernzerhof functional within the generalized gradient approximation method [4-5]. The core-valence interactions were accounted by the projected augmented wave (PAW) method [6]. The energy cutoff for plane wave expansions was set to 480 eV, and the 3×3×1 Monkhorst-Pack grid k-points were selected to sample the Brillouin zone integration. The structural optimization was completed for energy and force convergence set at 1.0×10^{-4} eV and 0.02 eV $Å^{-1}$, respectively.

The Gibbs free energy (G) can be calculated using the following formula:

$$G = H - TS$$

where G is the Gibbs free energy, H is the enthalpy of the system, T is the thermodynamic temperature (absolute temperature, in Kelvin, K), and S is the entropy of the system.

The Gibbs free energy changes (ΔG) of the reaction are calculated using the following formula:

$$\Delta G = \Delta E + \Delta ZPE - T\Delta S$$

where ΔE is the electronic energy difference directly obtained from DFT calculations, ΔZPE is the zero-point energy difference, T is the room temperature (298.15 K) and ΔS is the entropy change. Specifically, the formula for calculating the Gibbs free energy change value of $Fe_{1-x}S$ in LIBs can also be written as $\Delta G = G(Li_2S) + G(Fe) - G(Li) - G(Fe_{1-x}S)$. The four values on the right side of the Eq. are obtained by simulation calculation. Similarly, the Gibbs free energy change values for $Fe_{1-x}S$ in SIBs and PIBs can be calculated in the same equation. The barriers for Li/Na/K ion diffusion pathway were calculated with the climbing-image nudged elastic band (CI-NEB) method [7,8].

Supplementary Figures

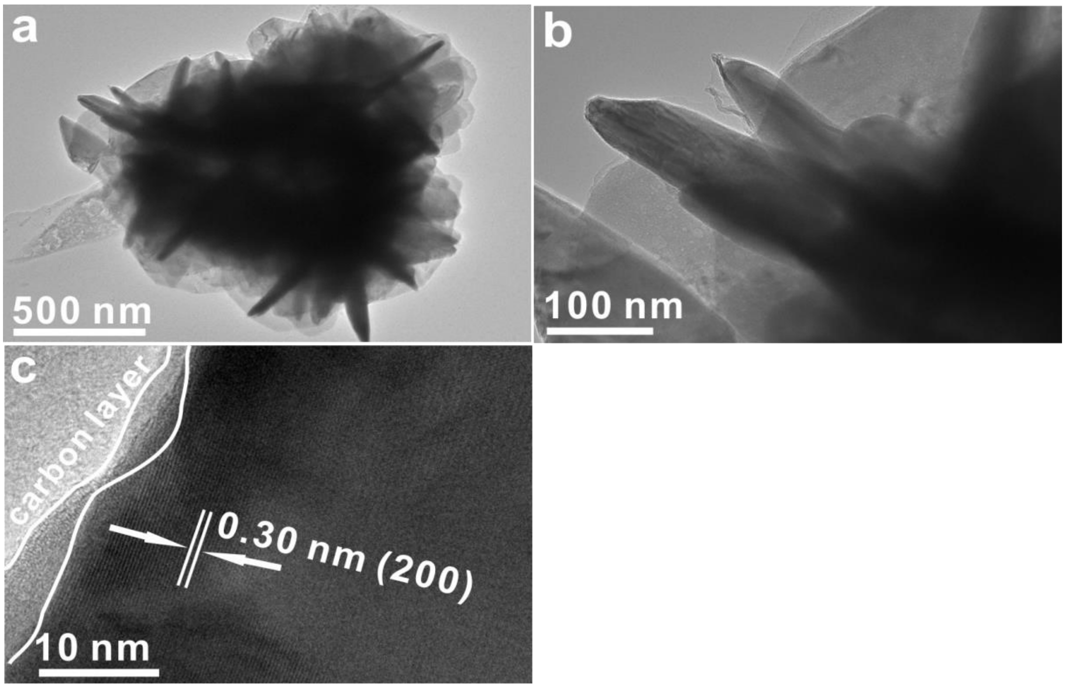

Figure S1. (a-c) TEM images of $Fe_{1-x}S/C$ nanosheets interwoven structure.

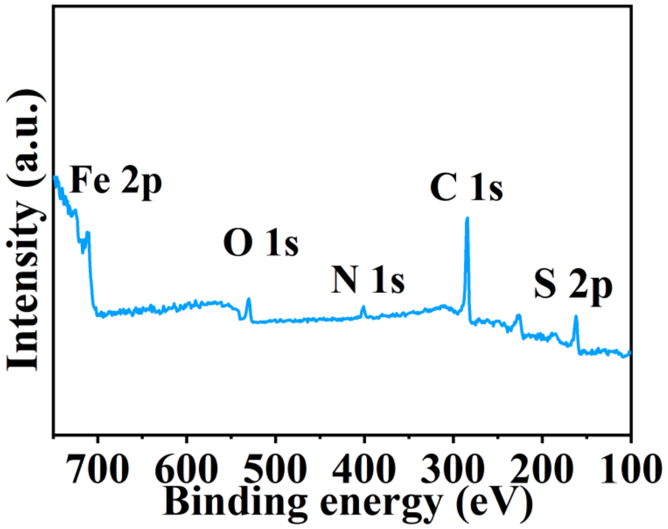

Figure S2. XPS survey spectrum of $Fe_{1-x}S/C$ nanosheets interwoven structure.

Table S1. Fitting result of XPS survey spectrum (Figure S2) of $Fe_{1-x}S/C$ nanosheets interwoven structure.

Sample	Fe (at%)	S (at%)	C (at%)	N (at%)	O (at%)
$Fe_{1-x}S/C$	19.48	21.64	53.92	1.62	3.34

Table S2 The elemental analysis results of the obtained samples.

Sample	Fe (wt%)	S (wt%)	C (wt%)	N (wt%)	O (wt%)
**$Fe_{1-x}S/C$**	58.268	36.997	4.427	0.183	0.125

The C, N, O, and S contents in the composites were measured using O/N/H and C/S elemental analyzers. The Fe contents in the composites can be calculated by the difference between 100 wt% and the total mass percentage of S/C/N/O. Inductively coupled plasma-atomic emission spectrometry (ICP-AES) was further used to determine the content of Fe, which is about 58.314wt%, basically consistent with the results of elemental analysis (Table S2). Based on the elemental analysis and ICP-AES results, it can be concluded that the atomic ratio of Fe and S is about 0.9, which is in accordance with the XPS result (Table S1).

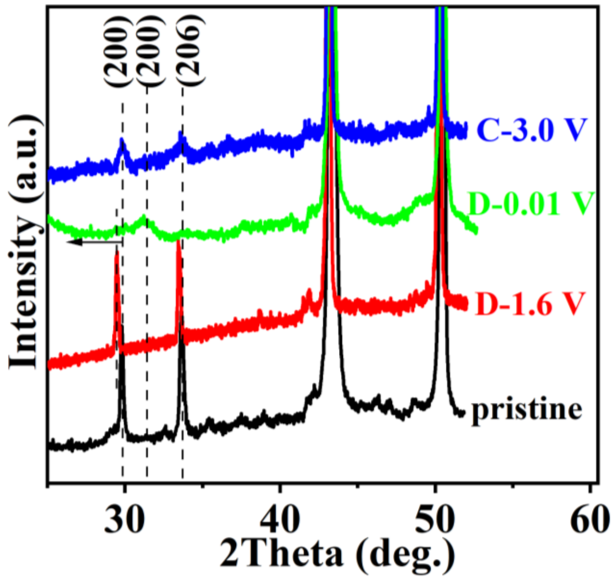

Figure S3. Ex-situ XRD patterns of $Fe_{1-x}S$/C electrode at different charge and discharge states in LIBs. D indicates discharge and C indicates charge.

When discharge to 1.5 V, the diffraction peaks of $Fe_{1-x}S$ slightly shift towards to a lower angle, which indicates the occurrence of insertion reaction. Obviously, after discharging to 0.01 V, the diffraction peaks of $Fe_{1-x}S$ totally disappear, indicating that the $Fe_{1-x}S$ nanosheet undergoes a complete conversion reaction during lithium storage. Furthermore, a new broad peak appears at 31.2° ascribed to (200) crystal plane of Li_2S (JCPDS: 23-0369). After charging to 3 V, the diffraction peaks of $Fe_{1-x}S$ reappear, indicating that the high reversibility of conversion reaction of $Fe_{1-x}S$ in LIBs.

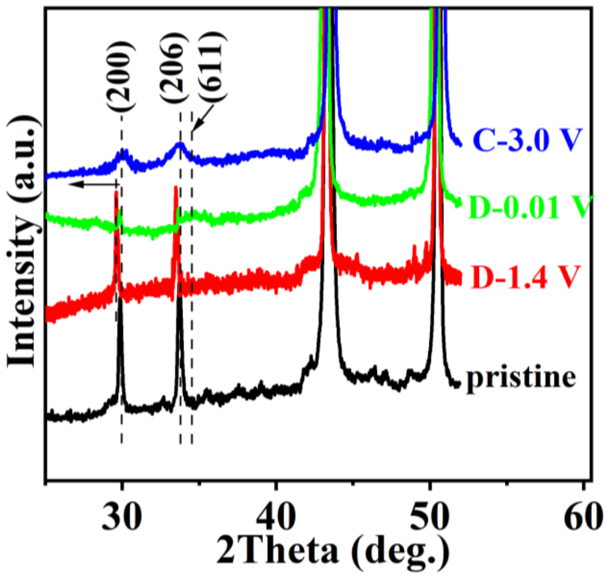

Figure S4. Ex-situ XRD patterns of $Fe_{1-x}S/C$ electrodes at different charge and discharge states in SIBs. D indicates discharge and C indicates charge.

When discharge to 1.4 V, the diffraction peaks of $Fe_{1-x}S$ slightly shift towards to a lower angle, which indicates the occurrence of insertion reaction. Obviously, after discharging to 0.01 V, the diffraction peaks of $Fe_{1-x}S$ still exist, indicating that the $Fe_{1-x}S$ nanosheet undergoes an incomplete conversion reaction during Na^+ storage. Furthermore, a new broad peak appears at ~34.7° ascribed to (611) crystal plane of Na_2S (JCPDS: 47-0178). After charging to 3 V, the diffraction peaks of $Fe_{1-x}S$ reappear, indicating that the high reversibility of conversion reaction of $Fe_{1-x}S$ in SIBs.

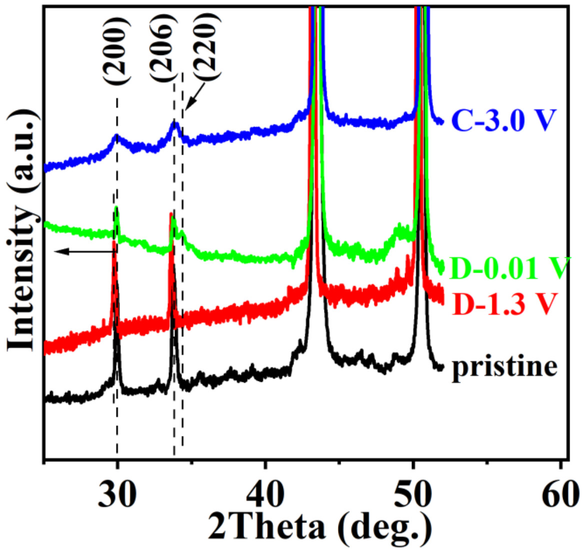

Figure S5. Ex-situ XRD patterns of $Fe_{1-x}S/C$ electrodes at different charge and discharge states in PIBs. D indicates discharge and C indicates charge.

When discharge to 1.3 V, the diffraction peaks of $Fe_{1-x}S$ slightly shift towards to a lower angle, which indicates the occurrence of insertion reaction. Obviously, after discharging to 0.01 V, the diffraction peaks of $Fe_{1-x}S$ still exist, indicating that the $Fe_{1-x}S$ nanosheet undergoes an incomplete conversion during K^+ storage. Furthermore, a new broad peak appears at ~34.3° ascribed to (220) crystal plane of K_2S (JCPDS: 47-1702). After charging to 3 V, the diffraction peaks of K_2S disappear, indicating that the high reversibility of conversion reaction of $Fe_{1-x}S$ in PIBs.

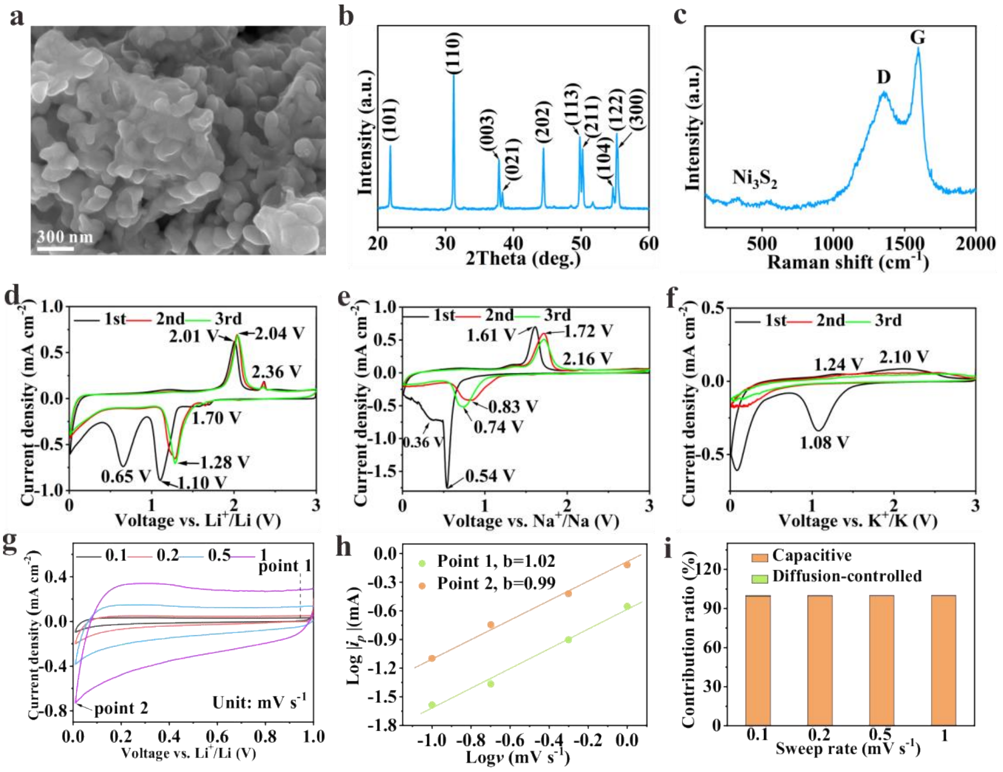

Figure S6. (a) SEM image, (b) XRD pattern, and (c) Raman spectrum of Ni_3S_2/C composites. (d-f) CV curves at 0.2 mV s^{-1} in LIBs (d), SIBs (e), and PIBs (f). (g-i) Kinetic analysis of Ni_3S_2/C electrodes from 0.01 to 1 V: (g) CV curves at different scanning rates, (h) Logi_p *vs* Log$v$, and (i) pseudocapacitive contribution percentages.

The sample was synthesized by hydrothermal method based on the previous report,[9] which shows nanoparticle morphology (Figure S6a). From XRD pattern (Figure S6b), the sample shows a series of diffraction peaks ascribed to (101), (110), (003), (021), (202), (113), (211), (104), (122), and (300) crystal planes of Ni_3S_2 (PDF#44-1418), confirming the formation of Ni_3S_2 crystals. From Raman spectrum (Figure S6c), two peaks appear at 1357 and 1590 cm^{-1} ascribed to D and G peaks of carbon materials, respectively, confirming the existence of carbon in the composite. Figure S6d shows the CV curves of Ni_3S_2 at 0.2 mV s^{-1} in LIBs. During the first cathodic scan, a weak peak at around 1.70 V can be indexed to the intercalation of Li^+ into the Ni_3S_2,[10] while the two cathodic peaks at around 1.10 and 0.65 V should be ascribed to the reduction of Ni_3S_2 to Ni and the formation of solid electrolyte interface (SEI) layer due to the

decomposition of electrolyte, respectively.[10] During the initial anodic scan, a sharp peak (2.01 V) and a weak peak (2.36 V) can be found, corresponding to the reformation of Ni_3S_2 after charging.[10] In the following scans, the cathodic peak shifts to 1.28 V. For the subsequent anodic scans, the sharp anodic peak is retained at around 2.04 V, while the weak peak similar to the first loop.[10] In Figure S6e for Ni_3S_2 in SIBs, during the first cathodic scan, the CV curves exhibit two peaks at 0.54 and 0.36 V attributed to the stepwise insertion and conversion reactions along with SEI formation, respectively.[11] In the initial charge scan, a clear oxidation peak at 1.61 V and a weak peak at 2.16 V are due to the oxidation of Fe/Na_2S.[11] In the following scans, the cathodic peak shifts to 0.83 and 0.74 V. As for the subsequent anodic scans, the sharp anodic peak is retained at around 1.72 V, while the weak peak is similar to the first loop.[11] In Figure S6f for Ni_3S_2 in PIBs, during the first cathodic scan, a strong and very broad reduction peak located at 1.08 V corresponds to the K-ion insertion, conversion reaction from Ni_3S_2 to K_2S and Ni, and the formation of the SEI layer.[12] In the initial charge scan, two oxidation peaks at 1.24 and 2.10 V can be assigned to the reversible conversion from K_2S/Ni to Ni_3S_2.[12] The CV curves of the second and third cycles have similar shapes and the same positions of redox peaks, indicating that the subsequent K-ion storage process has high reversibility. To comprehend the electrochemical kinetics and quantitative analysis of Ni_3S_2/C electrodes from V_{cc} to 0.01 V and from 0.01 V to Vos, we take LIBs as an example, the CV profiles from 0.01 to 1 V at various scan rates (0.1-1.0 mV s^{-1}) were measured. Apparently, the CV profiles exhibit rectangular shape during charge and discharge processes at different scanning rates (Figure S6g), which further indicates that capacitive-controlled process is dominant. The b-values of the marked points 1 and 2 in both cathodic and anodic processes are approximately equal to 1 (Figure S6h), which fully proves a complete capacitive-controlled process at the voltages of 0.01-1 V. As calculated in Figure S6i, the Ni_3S_2/C electrodes show ~100% capacitive contribution at various scanning rates.

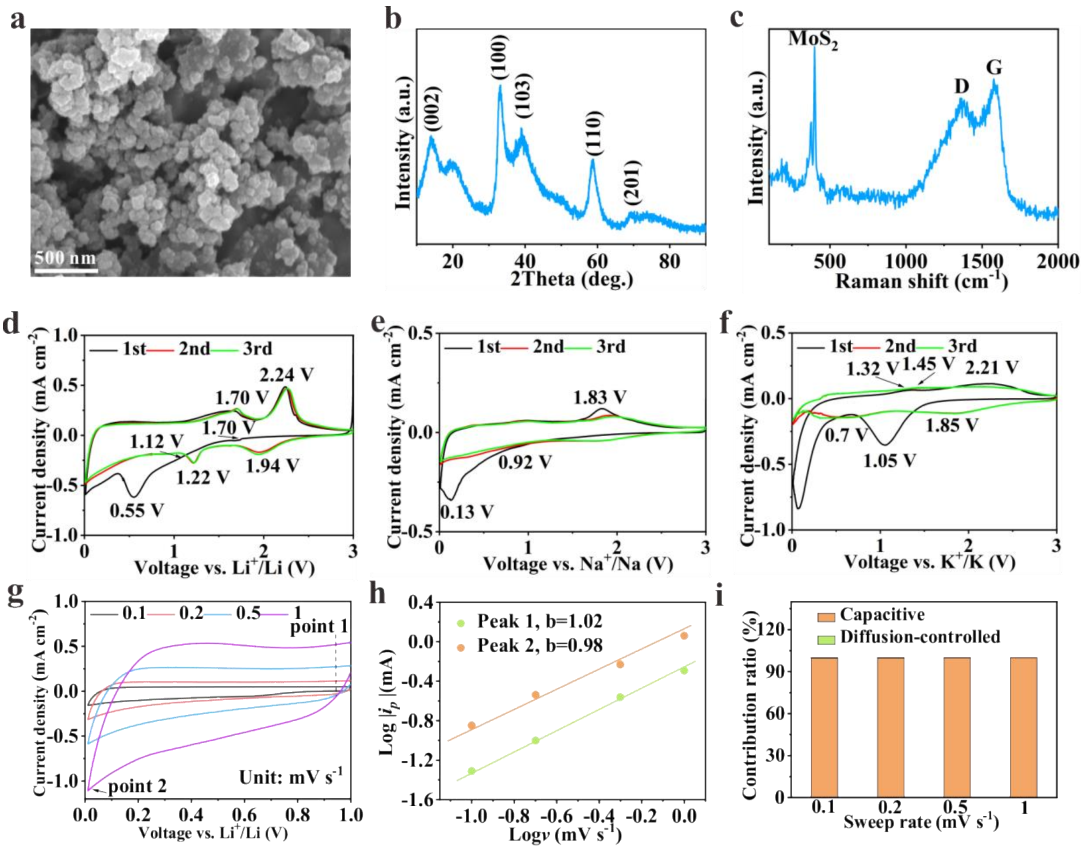

Figure S7. (a) SEM image, (b) XRD pattern, and (c) Raman spectrum of MoS_2/C: (d-f) CV curves at 0.2 mV s^{-1} in LIBs (d), SIBs (e), and PIBs (f). (g-i) Kinetic analysis of MoS_2/C electrodes from 0.01 to 1 V: (g) CV curves at different scanning rates, (h) Logi_p *vs* Log$v$, and (i) pseudocapacitive contribution percentages.

The sample was synthesized by a hydrothermal method based on the previous report,[13] which shows nanoparticle morphology (Figure S7a). From XRD pattern (Figure S7b), the sample shows a series of diffraction peaks ascribed to (002), (100), (103), (110), and (201) crystal planes of MoS_2 (PDF#37-1429), confirming the formation of MoS_2 crystals. From Raman spectrum (Figure S7c), two peaks appear at 1364 and 1576 cm^{-1} ascribed to D and G peaks of carbon materials, respectively, confirming the existence of carbon in the composite. Figure S7d shows the CV curves of MoS_2 in LIBs. In the first cathodic sweep, the two weak peaks at 1.70 and 1.12 V are ascribed to the Li-ion intercalation and conversion reaction.[14] A broad peak at 0.55 V can be seen, belonging to the formation of SEI layer.[14] In the following anodic sweep, a broad peak at 1.68 V

and a pronounced peak at 2.24 V are respectively attributed to the metal Mo oxidation and the conversion of Li_2S into S.[14] In the following cycles, the cathodic peaks corresponding to insertion and conversion reaction shift to the 1.94 and 1.22 V, respectively.[14] In Figure S7e for MoS_2 in SIBs, during the first cathodic scan, a peak appears at around 0.92 V, which is attributed to the insertion reaction.[15] The peak at 0.13 V originates from the conversion reaction.[15] During the first anodic scan, the peak at 1.83 V arises from the oxidation reaction of Mo to MoS_2.[15] In Figure S7f for MoS_2 in PIBs, the reduction peak (1.05 V) is associated with the formation of SEI and the K^+ insertion into MoS_2, ultimately generating Mo and K_2S in the conversion reaction.[16] In the initial oxidation scan, the broad oxidation peak of about 1.32 V belongs to the de-potassiation and oxidation of Mo to MoS_2 processes.[16] And the peak at 2.21 V is caused by the reversible conversion of K_2S into S.[16] The CV curves of the second and third cycles have similar shapes and the same positions of redox peaks, indicating that the subsequent potassium-ion storage process has high reversibility. To comprehend the electrochemical kinetics and quantitative analysis of MoS_2/C electrodes from V_{cc} to 0.01 V and from 0.01 V to Vos, we take LIBs as an example, the CV profiles from 0.01 to 1 V at various scan rates (0.1-1.0 mV s^{-1}) were measured. Apparently, the CV profiles exhibit rectangular shape during charge and discharge processes at different scanning rates (Figure S7g), which further indicates that capacitive-controlled process is dominant. The b-values of the marked points 1 and 2 in both cathodic and anodic processes are approximately equal to 1 (Figure S7h), which fully proves a complete capacitive-controlled process at the voltages of 0.01-1 V. As calculated in Figure S7i, the Ni_3S_2/C electrodes show ~100% capacitive contribution at various scanning rates.

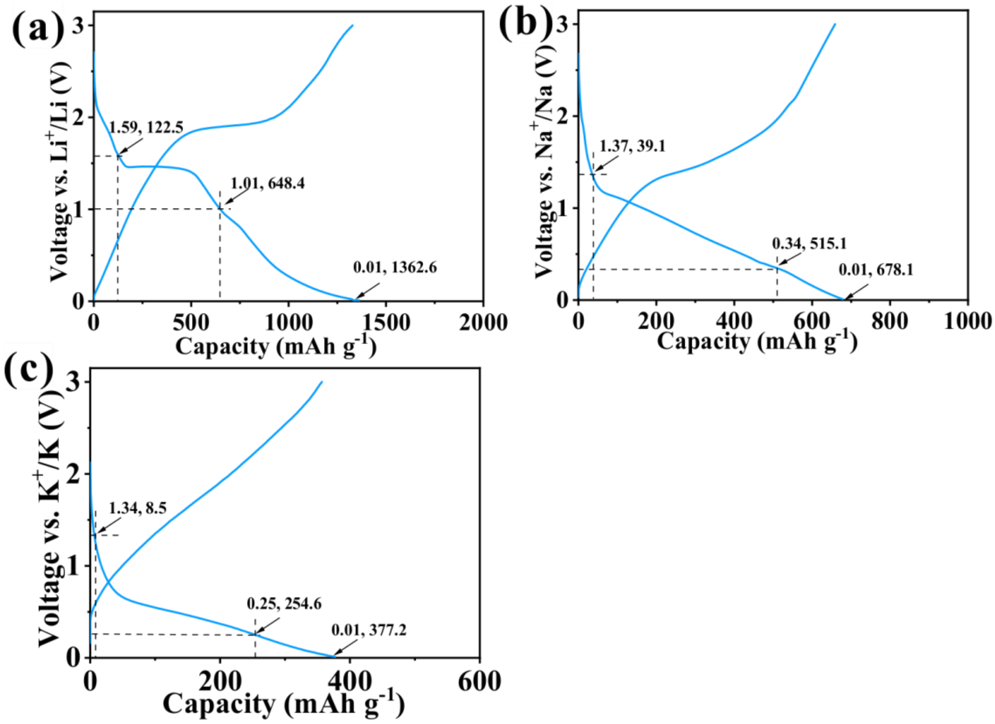

Figure S8. Charge and discharge curves in the second cycle in (a) LIBs, (b) SIBs, and (c) PIBs.

Table S3. The specific voltage range, capacity contribution, and capacity contribution ratio of insertion, conversion, and ion-electron decoupling of $Fe_{1-x}S$/C-700 electrodes in LIBs. The results are from in-situ magnetometry (Figure 5d) and charge/discharge curve (Figure S6a). open circuit voltage-OCV

Ion storage stages	Insertion	Conversion	Ion-electron decoupling
Voltage range (V)	OCV-1.59	1.59-1.01	1.01-0.01
Capacity contribution (mAh g^{-1})	122.5	525.9	714.2
Capacity contribution ratio (%)	9.0	38.6	52.4

Table S4. The specific voltage range, capacity contribution, and capacity contribution ratio of insertion, conversion, and ion-electron decoupling of $Fe_{1-x}S$/C-700 electrodes in SIBs. The results are from in-situ magnetometry (Figure 5e) and charge/discharge curve (Figure S6b). open circuit voltage-OCV

Ion storage stages	Insertion	Conversion	Ion-electron decoupling
Voltage range (V)	OCV-1.37	1.37-0.34	0.34-0.01
Capacity contribution (mAh g^{-1})	39.1	476.0	163.0
Capacity contribution ratio (%)	5.8	70.2	24.0

Table S5. The specific voltage range, capacity contribution, and capacity contribution ratio of insertion, conversion, and ion-electron decoupling of $Fe_{1-x}S$/C electrodes in PIBs. The results are from in-situ magnetometry (Figure 5f) and charge/discharge curve (Figure S6c). open circuit voltage-OCV

Ion storage stages	Insertion	Conversion	Ion-electron decoupling
Voltage range (V)	OCV-1.34	1.34-0.25	0.25-0.01
Capacity contribution (mAh g^{-1})	8.5	246.1	122.6
Capacity contribution ratio (%)	2.3	65.2	32.5

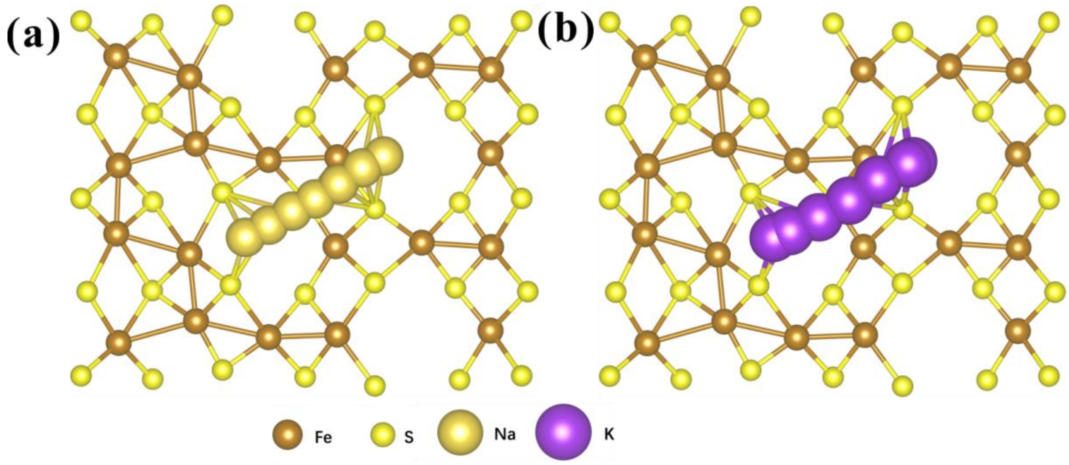

Figure S9. Top view of configurations of ion migration paths: (a) Na and (b) K.

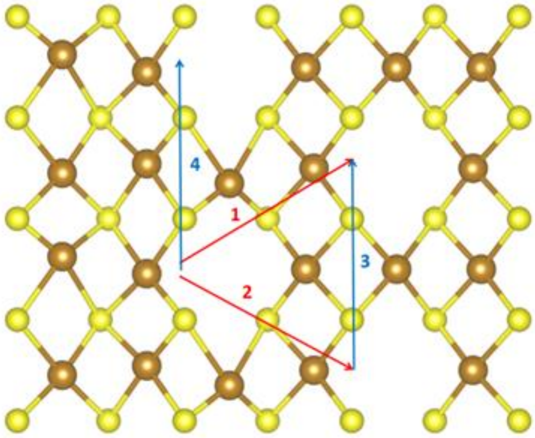

Figure S10. The possible ion migration paths in the top view of configurations.

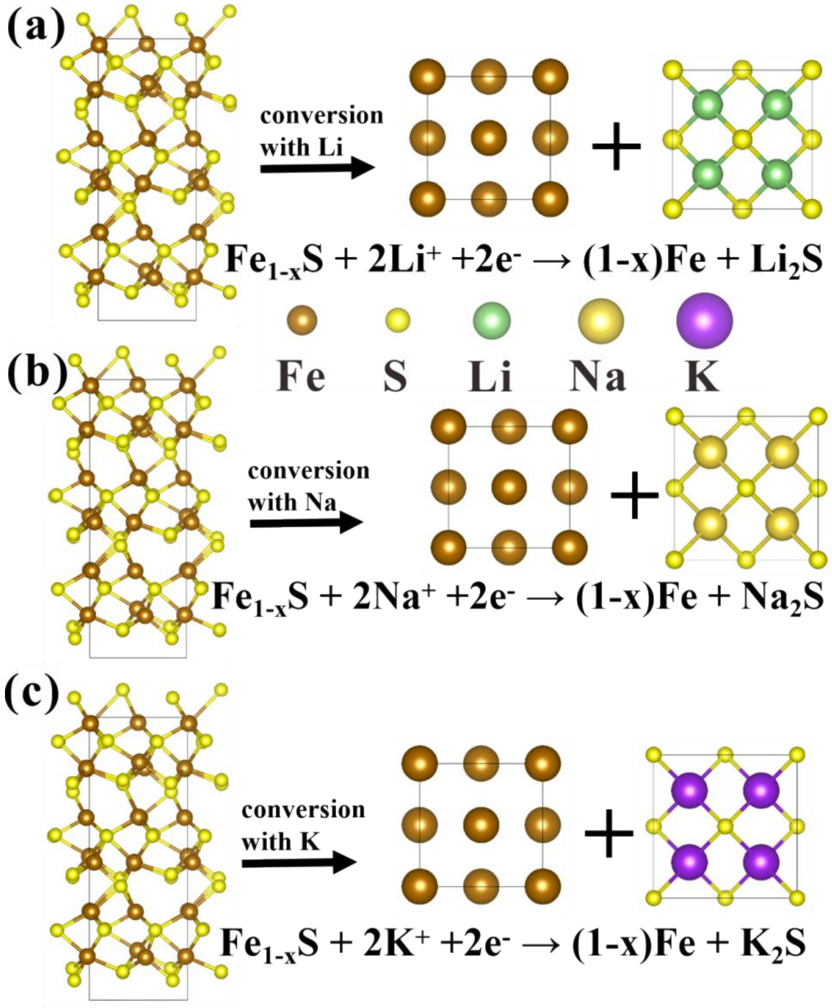

Figure S11. Atomic configurations in the calculation of the Gibbs free energy required for conversion reaction: (a) LIBs, (b) SIBs, and (c) PIBs.

Table S6. Magnetization of $Fe_{1-x}S/C$ electrodes in the second cycle from different voltage points in Figure 5d-f for LIBs, SIBs, and PIBs, respectively, and their corresponding magnetization values in these voltage points are marked as M_{Vx} (x=cc and os), where Vcc: Cut-off voltage of conversion reaction; Vos: Starting-voltage of oxidation reaction; $M_{Vcc-0.01}$: The magnetization difference value between M_{Vcc} and $M_{V0.01}$; $M_{Vos-0.01}$: The magnetization difference value between M_{Vos} and $M_{V0.01}$.

M_{Vx} (emu g^{-1})	M_{Vcc}	$M_{V0.01}$	M_{Vos}	$M_{Vcc-0.01}$	$M_{Vos-0.01}$
LIBs	69.3	42.3	86.2	27.0	43.9
SIBS	55.5	39.6	61.2	15.9	21.6
PIBs	37.7	37.5	38.1	0.2	0.6

References

[1] G. Kresse, and J. Hafner, *Phys. Rev. B* 1993, **47**, 558-561.

[2] G. Kresse, and J. Hafner, *Phys. Rev. B* 1994, **49**, 14251-14269.

[3] S. Gremme, J. Antony, S. Ehrlich, and H. Krieg, *J. Chem. Phys.* 2010, **132**, 154104.

[4] J. P. Perdew, K. Burke, and M. Ernzerhof, *Phys. Rev. Lett.* 1996, **77**, 3865-3868.

[5] G. Kresse, and D. Joubert, *Phys. Rev. B* 1999, **59**, 1758-1775.

[6] P. E. Blöchl, *Phys. Rev. B* 1994, **50**, 17953-17979.

[7] J. K. Nørskov, J. Rossmeisl, A. Logadottir, L. Lindqvist, J. R. Kitchin, T. Bligaard, and Jonsson H, *J. Phys. Chem. B* 2004, **108**, 17886-17892.

[8] L. I. Bendavid, and E. A. Carter, *J. Phys. Chem. C* 2013, **117**, 26048-26059.

[9] X. Guan, X. Liu, B. Xu, X. Liu, Z. Kong, M. Song, A. Fu, Y. Li, P. Guo, and H. Li, *Nanomaterials*, 2018, **8**, 760.

[10] J. Li, J. Li, Z. Ding, X. Zhang, Y. Li, T. Lu, Y. Yao, W. Mai, and L. Pan, *Chem. Eng. J.* 2019, **378**, 122108.

[11] G. D. Park, J. S. Cho, and Y. C. Kang, *NANOSCALE* 2015, **7**, 16721-16788.

[12] S. Zhang, F. Ling, L. Wang, R. Xu, M. Ma, X. Cheng, R. Bai, Y. Shao, H. Huang, D. Li, Y. Jiang, X. Rui, J. Bai, Y. Yao, and Y. Yu, *Adv. Mater.* 2022, **34**, 2201420.

[13] H. Qiu, H. Zheng, Y. Jin, M. Jia, Q. Yuan, C. Zhao, M. Jia, Ionics, 2020, **26**, 5543-5551.

[14] H. Zhang, Y. Liu, H. Jiang, Z. Deng, H. Liu, and C. Li, *Chem. Eng. Sci.* 2019, **207**, 611-618.

[15] M. Han, Z. Lin, and J. Yu, *J. Mater. Chem. A* 2019, **7**, 4804-4812.

[16] L. Chen, Z. Chen, L. Chen, P. Zhou, J. Wang, H. Yang, Z. Feng, X. Li, and J. Huang, *Appl. Surf. Sci.* 2023, **615**, 156253.
